# Effect of eicosapentaenoic and docosahexaenoic acid on resting and exercise-induced inflammatory and oxidative stress biomarkers: a randomized, placebo controlled, cross-over study

**DOI:** 10.1186/1476-511X-8-36

**Published:** 2009-08-19

**Authors:** Richard J Bloomer, Douglas E Larson, Kelsey H Fisher-Wellman, Andrew J Galpin, Brian K Schilling

**Affiliations:** 1 Cardiorespiratory/Metabolic Laboratory, Department of Health and Sport Sciences, The University of Memphis, Memphis, TN 38152, USA

## Abstract

**Background:**

The purpose of the present investigation was to determine the effects of EPA/DHA supplementation on resting and exercise-induced inflammation and oxidative stress in exercise-trained men. Fourteen men supplemented with 2224 mg EPA+2208 mg DHA and a placebo for 6 weeks in a random order, double blind cross-over design (with an 8 week washout) prior to performing a 60 minute treadmill climb using a weighted pack. Blood was collected pre and post exercise and analyzed for a variety of oxidative stress and inflammatory biomarkers. Blood lactate, muscle soreness, and creatine kinase activity were also measured.

**Results:**

Treatment with EPA/DHA resulted in a significant increase in blood levels of both EPA (18 ± 2 μmol·L^-1 ^vs. 143 ± 23 μmol·L^-1^; p < 0.0001) and DHA (67 ± 4 μmol·L^-1 ^vs. 157 ± 13 μmol·L^-1^; p < 0.0001), while no differences were noted for placebo. Resting levels of CRP and TNF-α were lower with EPA/DHA compared to placebo (p < 0.05). Resting oxidative stress markers were not different (p > 0.05). There was a mild increase in oxidative stress in response to exercise (XO and H_2_O_2_) (p < 0.05). No interaction effects were noted. However, a condition effect was noted for CRP and TNF-α, with lower values with the EPA/DHA condition.

**Conclusion:**

EPA/DHA supplementation increases blood levels of these fatty acids and results in decreased resting levels of inflammatory biomarkers in exercise-trained men, but does not appear necessary for exercise-induced attenuation in either inflammation or oxidative stress. This may be due to the finding that trained men exhibit a minimal increase in both inflammation and oxidative stress in response to moderate duration (60 minute) aerobic exercise.

## Background

Oxidative stress is a condition in which the production of reactive oxygen and nitrogen species (RONS) overwhelms the body's available antioxidant defenses, possibly resulting in oxidation within susceptible tissues [1]. This has been reported in response to both aerobic [2] and anaerobic [3] exercise, with over 300 original investigations published over the past 30 years [2]. While a low level of RONS production is necessary to maintain normal physiological function [4], as well as to allow for exercise-induced adaptations to the endogenous antioxidant defense system [5,6], excessive production of RONS can lead to the oxidation of lipids, proteins, and nucleic acids, potentially altering normal cellular function [7]. For example, acute and significant elevations in RONS may impair muscle force production [8], while more prolonged RONS production (possibly resulting from exercise overtraining) may impede exercise recovery [9]. Due to the association between increased RONS production and the pathogenesis of human disease, it has been an objective of some to regulate the oxidative stress response to acute exercise. Although somewhat controversial (See [10]), this is often done via the use of antioxidant nutrients.

In addition to RONS production and subsequent oxidative stress resulting from strenuous exercise, an often associated finding is acute and chronic inflammation [11-13]. In light of the relationship between RONS and inflammation, and the commonly measured biomarkers C-reactive protein (CRP) and tumor necrosis factor-alpha (TNF-α), inflammation is often increased in response to strenuous exercise [12-16]. In the short term, this appears to serve as a healing mechanism; however, *chronic *systemic inflammation (as with chronic systemic oxidative stress) has been associated with disease pathology [17]. Hence, methods of reducing systemic inflammation, such as the performance of regular physical activity [18], weight loss [19], and the use of dietary supplements [17] have been considered.

One class of nutrients that appears to possess both antioxidant and anti-inflammatory effects is essential fatty acids such as the fish oils eicosapentaenoic acid (EPA) and docosahexaenoic acid (DHA). While some reports have noted a decrease in resting oxidative stress and inflammatory biomarkers with EPA and DHA treatment [20-22], few studies have determined the effects of such supplementation in human subjects on exercise-induced changes in these measures [23-25]. In the few studies that have been conducted, results have been mixed, with issues such as the exercise protocol, test subjects (trained and untrained), dosage and duration of supplementation, the timing of measurement, and selection of biomarkers likely contributing to the discrepancies. Because so few studies investigating the effects of EPA/DHA supplementation have included exercise-trained individuals as research subjects, such individuals were used in the present study. This is because such dietary supplements are often promoted as being beneficial to these individuals for purposes of attenuating the oxidative stress and inflammatory response to exercise. It was our objective to determine if EPA/DHA supplementation is needed by exercise-trained individuals for these purposes.

Therefore, it was the purpose of this study to determine the effects of six weeks of EPA/DHA supplementation on resting and exercise-induced oxidative stress and inflammation in exercise-trained men. It was hypothesized that 1) EPA/DHA would result in a lowering of resting inflammatory and oxidative stress biomarkers, 2) that exercise would induce oxidative stress and inflammation following the use of both EPA/DHA and placebo, and 3) that EPA/DHA would attenuate the exercise-induced rise in these biomarkers, compared to placebo.

## Methods

### Subjects and screening

Only exercise-trained men were recruited to participate. Subjects were required to exercise both aerobically and anaerobically at least three days per week for a minimum of 30 minutes per session (for each type of exercise) for the past 12 months. In addition, subjects must have had an aerobic power (VO_2max_) of at least 40 mL·kg^-1^·min^-1^. Subjects were nonsmokers, not using anti-inflammatory or antioxidant agents, and did not report any history of cardiovascular or metabolic disorders.

Health history, drug and dietary supplement usage, and physical activity questionnaires were completed by all subjects to determine eligibility. Prior to participation, each subject was informed of all procedures, potential risks, and benefits associated with the study through both verbal and written form in accordance with the approved procedures of the University Institutional Review Board for Human Subjects Research. Potential recruits signed an informed consent form prior to being admitted as a subject.

Subjects' height, weight, and body composition via 7 site skinfold test and calculation using Siri equation was measured. Heart rate and blood pressure were recorded following a 10 minute period of quiet rest. Subject characteristics are shown in Table 1. It should be noted that of the 15 men included in the study, 13 were Caucasian and two were African American. A full explanation of dietary and physical activity data recording was provided to subjects, along with data collection forms. An overview of study procedures was also provided.

**Table 1:** Descriptive characteristics of 14 exercise-trained men

Variable	Value
Age (yrs)	25.5 ± 4.8
Height (cm)	174.4 ± 6.2
Weight (kg)	73.4 ± 7.7
Body fat (%)	10.0 ± 3.6
BMI (kg·m^-2^)	24.1 ± 1.6
Resting HR (bpm)	56 ± 8.7
Resting SBP(mmHg)	112.1 ± 9.8
Resting DBP(mmHg)	68.6 ± 7.2
VO_2max _(mL·kg^-1^·min^-1^)	47.0 ± 3.5
Years Aerobic Exercise	3.9 ± 3.6
Hours/wk Aerobic Exercise	4.4 ± 3.8
Years Anaerobic Exercise	6.3 ± 3.8
Hours/wk Anaerobic Exercise	5.3 ± 3.1

Data are mean ± SD.

A maximal graded exercise test (GXT) was conducted using a treadmill while expired gases were collected via facemask and analyzed using a SensorMedics Vmax 229™ metabolic system for determination of maximal oxygen consumption (VO_2max_). The collection and analysis of expired gases provided further descriptive characteristics regarding participants' aerobic power (e.g., VO_2max_). The test continued until volitional fatigue. Before and during the GXT, heart rate was continuously monitored via electrocardiograph (ECG) tracings using a SensorMedics Max-1™ ECG unit. Expired oxygen and respiratory exchange ratio data were continuously monitored via breath-by-breath samples. Blood pressure was monitored and the Borg scale of exertion was used to allow participants to indicate their level of perceived work. The highest 30 second average for VO_2 _was recorded as subjects' VO_2max_. Five subjects who underwent testing failed to reach the 40 mL·kg^-1^·min^-1 ^criteria. Therefore, these individuals were excluded from participation, leaving us with a total of 15 subjects entering randomization.

### Supplementation

**Figure 1:**
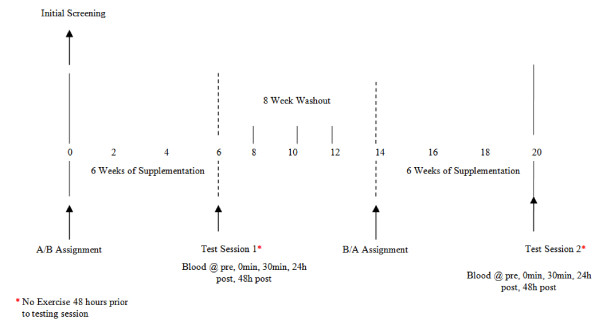
**Study timeline to investigate the effects of EPA/DHA on resting and exercise-induced inflammation and oxidative stress**.

The study design involved a random order cross-over assignment to EPA/DHA and placebo in a double blind manner. A schematic of the study timeline is presented in Figure 1. The total daily dosage of EPA (2224 mg; MorEPA Mini; Minami Nutrition, Belgium) and DHA (2208 mg; MorDHA Mini; Minami Nutrition, Belgium) was provided in eight gel capsules taken twice per day (morning and evening) with meals. The placebo gel capsules (taken in the same quantities) consisted of soybean oil and were identical in appearance, texture, and taste to the EPA/DHA capsules.

In both conditions, capsules were distributed to subjects by research assistants every two weeks in unlabeled boxes in amounts greater than needed for supplementation. Capsule counts upon box return allowed for estimation of compliance to intake.

### Acute exercise session

Following each six week period of EPA/DHA or placebo intake, subjects reported to the lab in the morning to perform a submaximal exercise test. Subjects were asked to walk on a treadmill while carrying a weighted backpack (weight equal to 25% of body mass) for 60 minutes. The treadmill speed and grade were altered every five minutes as outlined in Table 2. We chose to use a strenuous aerobic exercise test protocol which would result in an increase in RONS via increased oxygen consumption, in addition to other potential pathways (e.g., prostanoid metabolism, the autooxidation of catecholoamines, and oxidase enzymatic activity (NAD(P)H oxidase, xanthine oxidase [26]). Rather than simply use a traditional steady state aerobic exercise protocol which would result in a moderate increase in oxygen consumption, we attempted to mimic a strenuous uphill climb, in which individuals perform intermittent exercise while carrying an excess load. The addition of the extra load would theoretically increase the amount of work performed, resulting in a greater increase in oxygen consumption and subsequent RONS generation via mitochondrial electron leakage. We chose such a protocol as many previous studies using exercise-trained individuals involved in moderate duration aerobic exercise have noted minimal increase in oxidative stress and inflammatory biomarkers [2]. Of course, we could have also opted to use strenuous and intermittent eccentric resistance exercise as our chosen stressor, as such exercise often results in acute and chronic oxidative stress and inflammation. However, our recent findings for this form of exercise (eccentric multi-joint) indicate only minimal increases in both oxidative stress and inflammatory biomarkers-when using resistance trained men as research subjects [27]. Therefore, based on these findings of only minimal increase in oxidative stress and inflammation when using exercise trained subjects performing traditional aerobic or eccentric resistance exercise, we opted to use an aerobic exercise model with an exaggerated external load in the present design. Pilot testing was found to result in a significant physical stress, much more so than traditional aerobic or resistance exercise.

**Table 2:** Exercise data from 14 exercise-trained men during 60 minutes of treadmill walking

Ending Minute →	5	10	15	20	25	30	35	40	45	50	55	60
Speed (mph)	3.5	3.5	3.5	3.5	3.5	3.5	3.5	3.5	3.5	3.0	3.0	3.5
Grade (%)	0	5	10	0	5	10	0	5	10	12.5	0	15

Heart Rate (bpm) *EPA/DHA*	104.09 ± 3.46	130.42 ± 3.79	159.67 ± 5.10	122.33 ± 4.06	140.17 ± 5.34	168.33 ± 4.93	128.25 ± 4.77	148.50 ± 5.32	168.73 ± 4.85	166.64 ± 4.23	129.73 ± 3.86	183.20 ± 3.74
Heart Rate (bpm) *Placebo*	107.18 ± 4.38	130.33 ± 4.79	161.42 ± 4.98	122.42 ± 4.23	144.42 ± 4.92	169.00 ± 4.98	131.91 ± 5.33	150.42 ± 5.80	170.09 ± 5.60	167.55 ± 5.23	130.82 ± 5.06	180.00 ± 3.83
RPE *DHA/EPA*	9.67 ± 0.56	12.00 ± 0.44	15.15 ± 0.46	11.62 ± 0.45	14.08 ± 0.56	16.62 ± 0.49	12.69 ± 0.49	14.23 ± 0.67	16.92 ± 0.45	15.75 ± 0.54	12.42 ± 0.60	19.18 ± 0.23
RPE *Placebo*	9.18 ± 0.67	11.77 ± 0.50	14.69 ± 0.43	11.85 ± 0.42	13.54 ± 0.37	16.23 ± 0.43	11.38 ± 0.58	14.00 ± 0.48	16.58 ± 0.31	15.83 ± 0.55	11.67 ± 0.62	19.40 ± 0.22
VO_2_ (L·min^-1^) *EPA/DHA*	1.00 ± 0.12	1.81 ± 0.15	2.12 ± 0.15	1.23 ± 0.09	1.56 ± 0.16	1.91 ± 0.22	1.17 ± 0.11	1.57 ± 0.15	2.00 ± 0.13	1.86 ± 0.13	1.39 ± 0.19	2.35 ± 0.21
VO_2_ (L·min^-1^) *Placebo*	0.98 ± 0.09	1.39 ± 0.14	1.98 ± 0.14	1.12 ± 0.18	1.61 ± 0.17	1.89 ± 0.15	1.15 ± 0.16	1.65 ± 0.15	2.01 ± 0.12	1.79 ± 0.11	1.16 ± 0.27	2.43 ± 0.20
RER *EPA/DHA*	0.91 ± 0.01	0.97 ± 0.02	1.02 ± 0.02	1.00 ± 0.02	0.96 ± 0.03	1.00 ± 0.02	0.93 ± 0.01	0.96 ± 0.03	0.98 ± 0.02	0.96 ± 0.02	0.91 ± 0.02	1.08 ± 0.04
RER *Placebo*	0.89 ± 0.01	0.95 ± 0.01	1.02 ± 0.02	0.94 ± 0.02	0.93 ± 0.03	1.02 ± 0.03	0.99 ± 0.04	0.90 ± 0.02	1.02 ± 0.03	1.00 ± 0.04	0.95 ± 0.03	1.09 ± 0.03

Data are mean ± SEM.

No statistically significant differences noted (p > 0.05).

RPE = rating of perceived exertion; RER = respiratory exchange ratio

Because our climb was similar to that performed by the military in a typical physical march, we consulted with a Marine Corps ROTC Captain, in addition to information reported in the United States Army Infantry Combat School handbook . This allowed for determination of the test duration and pack load. The specific speed and grade of each stage of testing was determined through the same consultation, in addition to pilot testing. Breath samples were collected from subjects during the last two minutes of each five minute stage throughout the exercise test for analysis of expired gases (SensorMedics Vmax 229™ metabolic system). In addition, heart rate and perceived exertion (6-20 Borg scale) were recorded. Subjects completed the identical exercise test following each six week period of EPA/DHA or placebo intake. Although subjects performed the test in the morning following an eight hour overnight fast, they were allowed to drink water ad libitum before, during, and following the exercise test. Water intake was matched for both exercise test days.

### Blood sampling

Venous blood samples (~20 mL) were taken from subjects' forearm via needle and Vacutainer™. Blood samples were collected pre and post intervention for both EPA/DHA and placebo conditions, and analyzed for plasma EPA and DHA concentrations using Gas Chromatography-Flame Ionisation Detection (GC-FID). In relation to the exercise test, blood samples were collected pre exercise (following a 10 minute quiet rest), 0 hours post-exercise, 0.5 hours post-exercise, and 24 and 48 hours post-exercise. Following collection, blood samples were processed accordingly, and the plasma/serum was immediately stored at -80°C until analyzed. As markers of inflammation, CRP was analyzed in serum using an ultra-sensitive enzyme linked immunosorbent assay (ELISA) procedure as described by the manufacturer (Diagnostic Systems Laboratories, Webster, TX) and TNF-α was analyzed in plasma using an ELISA procedure as described by the manufacturer (Caymen Chemical, Ann Arbor, MI). Antioxidant capacity was analyzed in serum using the Trolox-equivalent antioxidant capacity (TEAC) assay using procedures outlined by the reagent provider (Sigma Chemical, St. Louis, MO). As markers of oxidative stress, we selected a wide array of commonly studied variables in order to best characterize the system. Protein carbonyls were analyzed in plasma using an ELISA procedure as described by the manufacturer (Zenith Technologies, Dunedin, NZ). Serum titers of IgG-autoantibodies against oxidized low density lipoprotein (LDL) were analyzed using an ELISA procedure as described by the manufacturer (OLAB, Biomedica). Malondialdehyde was analyzed in plasma using a commercially available colorimetric assay (Northwest Life Science Specialties, Vancouver, WA), using the modified method described by Jentzsch et al. [28]. Hydrogen peroxide and xanthine oxidase activity were analyzed in plasma using the Amplex Red reagent method as described by the manufacturer (Molecular Probes, Invitrogen Detection Technologies, Eugene, OR). Nitric oxide was estimated using the nitrate/nitrite assay procedure (Caymen Chemical, Ann Arbor, MI). Whole blood lactate (pre, 0 hours, 0.5 hours only) was analyzed using an automated unit (Accutrend; Roche Diagnostics, Mannheim, Germany).

In order to determine any degree of muscle injury, the common indirect markers of injury including creatine kinase activity and muscle soreness were chosen. Creatine kinase activity was measured spectrophotometrically using commercially available reagents (StanBio Labs, Boerne, TX). Muscle soreness was assessed using a 10 cm visual analog scale where "0" represents no pain and "10" represents intense pain [29]. Subjects reported their perceived muscle soreness following body-weight squatting (two repetitions). This was done at all blood collection time points.

### Dietary and physical activity records

All subjects were instructed to maintain their normal diet, and record their food and beverage intake during the seven day period prior to each exercise test day. Nutritional records were analyzed for total calories, protein, carbohydrate, fat, and a variety of micronutrients (Food Processor SQL, version 9.9, ESHA Research, Salem, OR). Subjects were given specific instructions regarding abstinence of alcohol consumption during the 48 hours immediately preceding the test days. Subjects were instructed to maintain their normal physical activity, with the exception of refraining from activity during the 48 hours preceding and following each test day.

### Statistical analysis

For the main analysis, all dependent variables were analyzed using a 2 (group) × 5 (time) repeated measures analysis of variance (ANOVA). Blood EPA and DHA data were analyzed using a 2 (group) × 2 (time) ANOVA. Significant interactions and main effects were further analyzed using Tukey's *post hoc *tests. Dietary and physical activity data were analyzed using a t-test. All analyses were performed using JMP statistical software (version 4.0.3, SAS Institute, Cary, NC). Statistical significance was set at p = 0.05. The data are presented as mean ± SEM, except for subject descriptive characteristics (mean ± SD).

## Results

Of the 15 subjects who were enrolled in the study, one was dropped due to failure to complete the second exercise test (due to injury unrelated to the study). Therefore, only 14 subjects' data are included in the analyses. Regarding compliance to capsule intake, subjects were 91% compliant to EPA/DHA capsules and 97% compliant to placebo capsules, with no statistical difference noted (p = 0.276). No difference was noted between conditions in subjects' dietary intake for total kilocalories, total grams of protein, percentage protein, total carbohydrate, percentage carbohydrate, total fat, percentage fat, vitamin C intake, vitamin E intake, or vitamin A intake (p > 0.05). Data are presented in Table 3.

**Table 3:** Dietary data of 14 exercise-trained men during the 7 days preceding exercise testing

Variable	EPA/DHA	Placebo
Kcal	2603 ± 209	2692 ± 217
Protein (g)	123 ± 9	130 ± 11
% Protein	20 ± 2	20 ± 3
CHO (g)	348 ± 33	355 ± 38
% CHO	53 ± 2	53 ± 3
Fat (g)	84 ± 9	84 ± 11
% Fat	29 ± 1	28 ± 2
Vitamin C (mg)	100 ± 24	144 ± 29
Vitamin E (mg)	13 ± 4	8 ± 3
Vitamin A (RE)	4448 ± 566	5113 ± 586

Data are mean ± SEM.

No statistically significant differences noted (p > 0.05).

### Exercise test data

No difference was noted between conditions for heart rate, perceived exertion, VO_2_, or respiratory exchange ratio (p > 0.05). Data are presented in Table 2. Blood lactate was increased at 0 and 0.5 hours post exercise compared to pre exercise (p < 0.05), but not different (p > 0.05) between EPA/DHA (pre: 1.78 ± 0.31; 0 post: 6.33 ± 0.43; 30 post: 2.86 ± 0.33 mmol·L^-1^) and placebo (pre: 1.86 ± 0.33; 0 post: 6.20 ± 0.46; 30 post: 2.76 ± 0.32 mmol·L^-1^).

### Inflammatory and oxidative stress data

Treatment with EPA/DHA resulted in a significant increase in blood levels of both EPA (18 ± 2 μmol·L^-1 ^vs. 143 ± 23 μmol·L^-1^; p < 0.0001) and DHA (67 ± 4 μmol·L^-1 ^vs. 157 ± 13 μmol·L^-1^; p < 0.0001). No differences were noted for placebo in EPA (34 ± 9 μmol·L^-1 ^vs. 28 ± 5 μmol·L^-1^; p > 0.05) and DHA (98 ± 12 μmol·L^-1 ^vs. 86 ± 7 μmol·L^-1^; p > 0.05).

**Figure 2:**
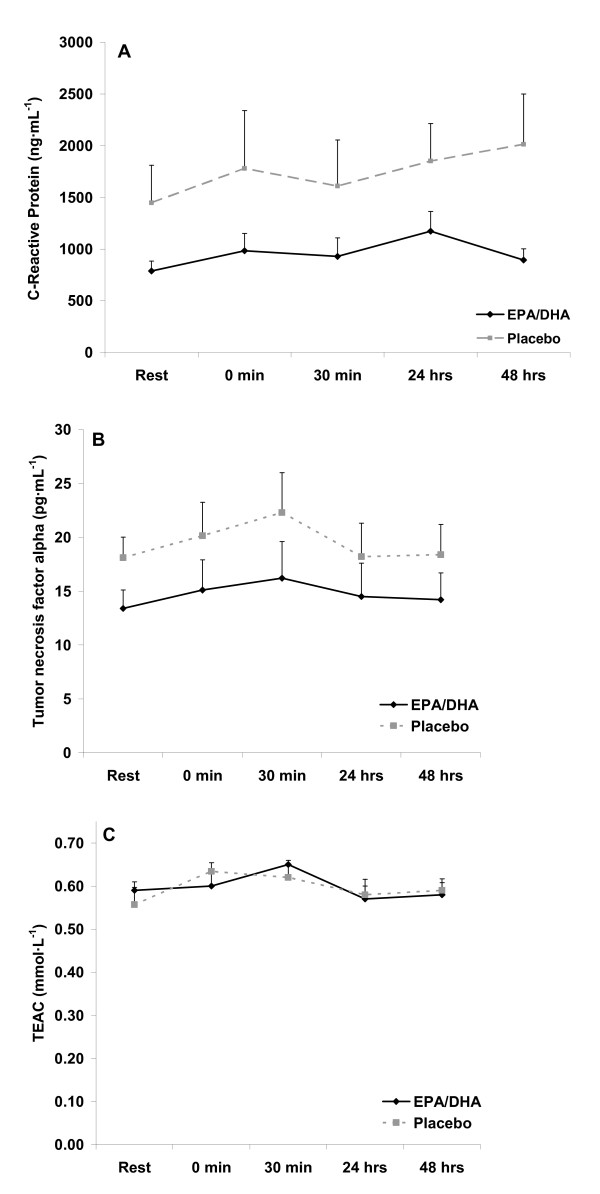
**Blood C-reactive protein (A), tumor necrosis factor-alpha (B), and trolox equivalent antioxidant capacity (C) in exercise-trained men before and following 60 minutes of treadmill exercise**.

**Figure 3:**
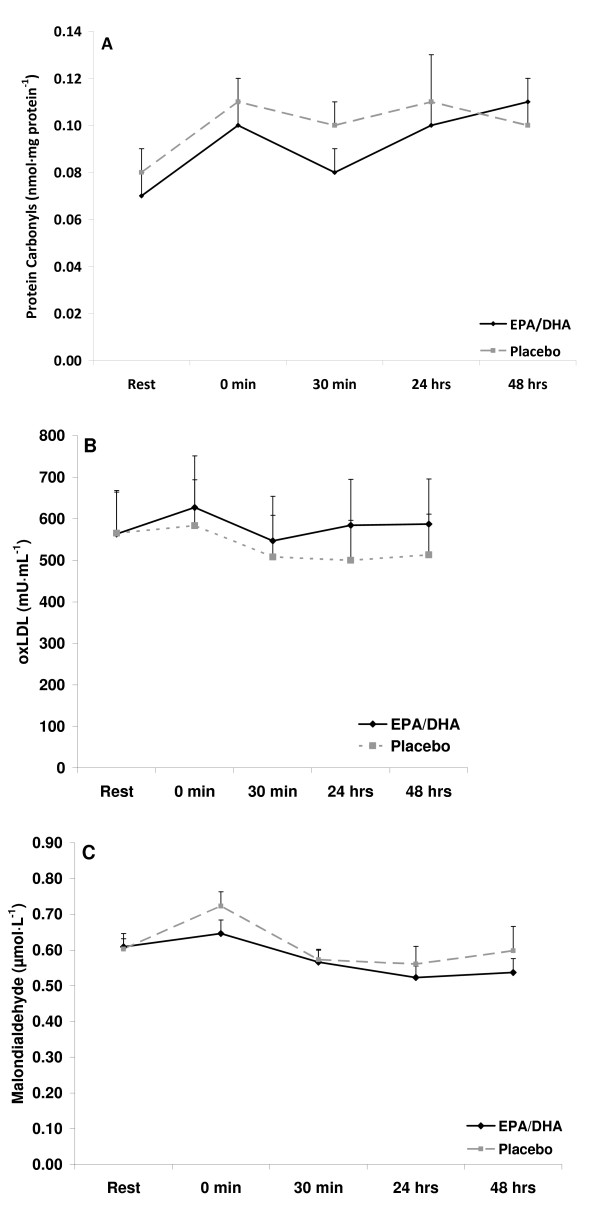
**Blood protein carbonyls (A), oxidized low density lipoprotein (B), and malondialdehyde (C) in exercise-trained men before and following 60 minutes of treadmill exercise**.

**Figure 4:**
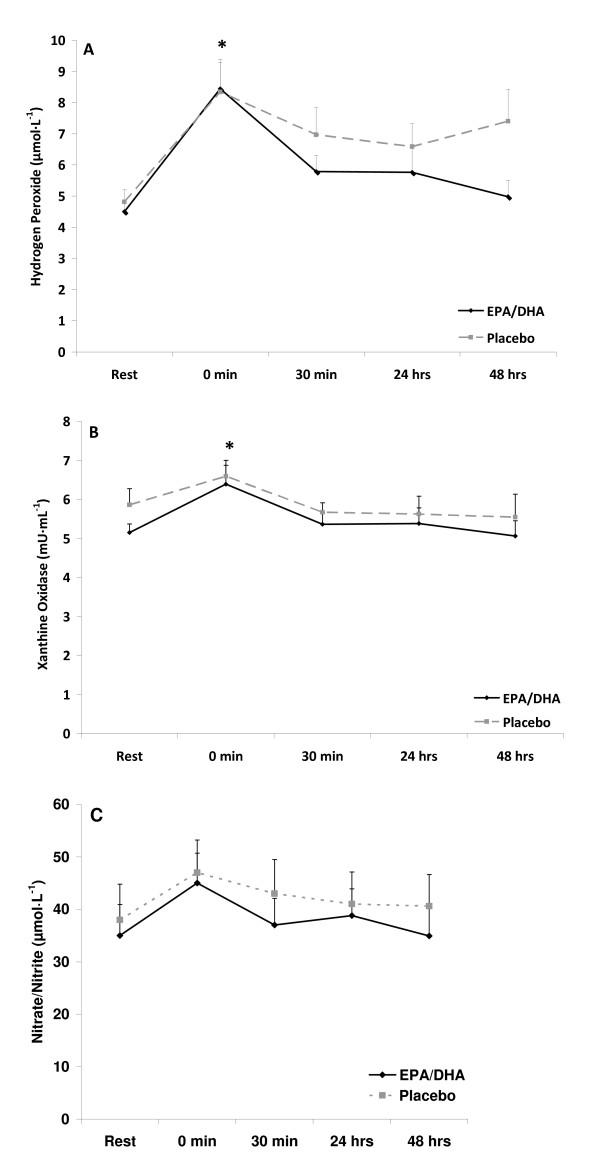
**Blood hydrogen peroxide (A), xanthine oxidase activity (B), and nitrate/nitrite (C) in exercise-trained men before and following 60 minutes of treadmill exercise**.

Resting levels of CRP and TNF-α were lower with EPA/DHA compared to placebo (p < 0.05). However, resting oxidative stress markers were not different (p > 0.05). There was an increase in oxidative stress in response to exercise (time effect with regards to XO [p = 0.032] and H_2_O_2 _[p = 0.0001]), with values for both of these variables higher at 0 hours post exercise compared to pre exercise (p < 0.05). No other time effects were noted. In addition, no interaction effects were noted, perhaps due to the relatively minor increase noted in these variables. However, a condition effect was noted for both CRP (p < 0.0003) and TNF-α (p < 0.05), with lower values with the EPA/DHA condition. No other condition effects were noted. Inflammatory and oxidative stress biomarkers measured before and after exercise are presented in Figures 2, 3, and 4.

### Muscle injury data

Creatine kinase activity was not statistically increased with exercise (p > 0.05), and very similar for both EPA/DHA (56 ± 8, 91 ± 13, 74 ± 11, 95 ± 14, 70 ± 16 U·L^-1^) and placebo (66 ± 12, 90 ± 16, 75 ± 13, 87 ± 17, 89 ± 13 U·L^-1^) at pre, 0, 0.5, 24, and 48 hours post exercise, respectively. A time main effect was noted for muscle soreness (p = 0.0003) with 0 and 0.5 hours post-exercise higher than rest (p = 0.05). No interaction effect was noted (p > 0.05), with near identical values for EPA/DHA (0 ± 0, 3.9 ± 0.5, 2.5 ± 0.3, 1.8 ± 0.3, 1.2 ± 0.1) and placebo (0 ± 0, 3.78 ± 0.5, 3.0 ± 0.4, 2.3 ± 0.4, 1.7 ± 0.3) at pre, 0, 0.5, 24, and 48 hours post exercise, respectively.

## Discussion

Findings from the present investigation indicate that EPA/DHA supplementation at a daily dosage of 2224 mg EPA+2208 mg DHA for 6 weeks 1) significantly increases blood levels of these fatty acids, 2) results in decreased resting levels of inflammatory biomarkers, but 3) does not appear necessary for attenuation of exercise-induced oxidative stress or inflammation in a sample of exercise-trained men. This latter finding may be due to the observation that trained men exhibit a minimal increase in both inflammation and oxidative stress in response to a moderate duration, aerobic exercise.

We chose to use an aerobic exercise protocol of moderate duration in order to mimic a type and duration of exercise commonly performed by many individuals. We included an exaggerated external load component in our protocol (use of a weighted backpack) in order to increase the metabolic demands of the exercise, which theoretically would have increased RONS production and subsequent oxidative stress. Previous studies using exercise-trained subjects performing moderate duration aerobic or anaerobic exercise bouts have largely resulted in minimal increase in oxidative stress [2], likely due to the upregulation in endogenous antioxidant protective mechanisms within such individuals [30]. With this knowledge, we did not want to use such an exercise protocol, with the probability of observing an oxidative stress response being minimal. Hence, we decided to include a moderate duration aerobic exercise bout with the additional load, in order to increase the metabolic demands. To our knowledge, no study to date has used such a protocol in an attempt to elicit an oxidative stress response. Our findings of a lack of such response within our sample of exercise-trained men provide additional evidence that individuals who engage in regular, strenuous exercise experience minimal oxidative stress in response to moderate duration aerobic exercise bouts, even when such bouts are strenuous in nature (as evidenced by our blood lactate, heart rate, and perceived exertion data-Table 2).

Due to these observations, we conclude that supplemental antioxidant/anti-inflammatory agents such as EPA/DHA are not necessary for such individuals, as the oxidative stress and inflammatory response is minimal. There is simply no additional need for such exogenous protection. This does not indicate, however, that use of EPA/DHA may not prove beneficial for either untrained individuals who are beginning an exercise program (whether for recreation or therapeutic reasons), or for individuals engaged in longer duration exercise involving extensive muscle injury. Such situations may lead to increased RONS formation, inflammation, and muscle injury. Under these conditions, supplemental EPA/DHA may prove beneficial-based on our findings of decreased CRP and TNF-α. Of course, further research is necessary to verify this hypothesis.

Oxidative stress has been reported to be elevated in response to acute exercise in several investigations, although this is not always the case [2]. The findings for increased oxidative stress are relatively uniform when untrained subjects are used within the research design. Of course, the downside to such work is that the results cannot be generalized to a population of exercise-trained subjects. Considering that dietary supplements for purposes of muscle recovery following exercise are typically marketed to individuals engaged in regular exercise, studies including untrained individuals may provide little information as it relates to the target population. For this reason, trained subjects were selected for this experiment.

In contrast to the findings for increased oxidative stress in response to exercise in untrained subjects, several studies using exercise-trained individuals have reported minimal increase in exercise-induced oxidative stress, when exercise bouts are of moderate duration (= 60 minutes) and intensity (= 70% VO_2max_) [2]. More consistent findings of elevated oxidative stress in exercise-trained subjects are available for long duration exercise bouts, often involving marathon or triathlon competition [2]. It is possible that antioxidant/anti-inflammatory supplements may be beneficial for such individuals during these times, as some studies have reported attenuation in exercise-induced oxidative stress/inflammation with use of such agents [15,25,31-35]. Considering that excessive oxidative stress [7] and inflammation [17] has been linked to the development and progression of several chronic diseases, use of agents in an attempt to minimize (but not eliminate) increases in both oxidative stress and inflammation may be reasonable. This is especially true for individuals who are routinely involved in *excessive *strenuous exercise, as such an activity pattern has been associated with ill-health and premature death [36]. However, specific data addressing this issue are certainly needed.

Considering the above, coupled with the findings of the present study, it is possible that our chosen exercise protocol was of too low an intensity and duration to promote a significant oxidative stress, in particular within our sample of exercise-trained men. The production of RONS has been reported to increase in an intensity [37,38] and duration dependent manner [39]. Therefore, we may have observed a greater magnitude of increase in our chosen biomarkers if the exercise bout was extended in duration and was more intense. That being said, it should be understood that increasing either of these variables would have made it extremely difficult for subjects to complete the protocol. Most were visibly exhausted at the conclusion of testing, and commented on the extreme difficulty of the protocol. We believe that our findings confirm previous reports which indicate that even with strenuous exercise of moderate duration; individuals who are exercise-trained experience minimal increase in exercise-induced oxidative stress and inflammation. This lack of response is likely one of the additional and often overlooked benefits of regular exercise training; that is, an upregulation in endogenous antioxidant defense mechanisms [6,30,40]. These findings have applicability to most individuals who include exercise as a component of their daily routine. Specifically, only a small percentage of exercise enthusiasts typically train for longer than one hour per session. Our findings have specificity towards such individuals, even when the exercise training is rather challenging.

Given that the chosen protocol was concentric in nature, the amount of muscle damage (and subsequent related RONS production) would be expected to be minimal. This was confirmed by our findings of insignificant increases in muscle soreness and creatine kinase activity in blood. Our results may have been different if using either an aerobic exercise bout involving an eccentric bias (e.g., downgrade running) or a heavy resistance exercise protocol involving a great degree of eccentric muscle actions. However, as mentioned earlier, our recent findings using trained men performing eccentric multi-joint resistance exercise indicate only a minimal increase in both oxidative stress and inflammatory biomarkers [27]. Therefore, we opted to use an aerobic exercise model with an exaggerated external load in an attempt to stimulate a further increase in RONS via increased mitochondrial electron leakage.

As with oxidative stress, prior research has shown that the exercise-induced inflammatory response is intensity [11] and duration [41] dependent. In the same way that our protocol may have been of too low an intensity and duration to significantly increase oxidative stress biomarkers, this may have also been true for CRP and TNF-α. This is underscored by the finding of Mastaloudis et al. [14], who reported elevated CRP, TNF-α, and interleukin-6 (IL-6) in athletes performing a 50 km ultra-marathon. TNF-α and IL-6 were elevated significantly as early as mid-race, while CRP reached significance in the hours following the race, peaking at 24 hours, and remaining 2 fold higher than baseline at the 6^th ^day of recovery. These findings are in contrast to those of the present study in which CRP and TNF-α were not altered in a statistically significant manner, even through 48 hours into recovery.

## Conclusion

Supplementation with EPA/DHA increases blood levels of these fatty acids and is associated with a decrease in resting CRP and TNF-α. However, EPA/DHA supplementation does not appear necessary for attenuation of exercise-induced oxidative stress or inflammation in a sample of exercise-trained men. Such individuals experience a minimal increase in oxidative stress and inflammation in response to a moderate duration, aerobic exercise. Hence, EPA/DHA supplementation should not be specifically recommended for this purpose. Future studies investigating the effect of antioxidant/anti-inflammatory agents within a sample of exercise-trained subjects should consider using a protocol of longer duration, and possibly higher intensity, in order to produce a more robust oxidative stress response. This may allow for a better assessment of the effectiveness of such nutrient intervention. As our data show, EPA/DHA intake at the dosage and duration provided in the present study may serve to combat systemic inflammation in a sample of exercise-trained men. These findings may have implications for those who train intensively, as well as other individuals who are prone to inflammatory conditions, as use of EPA/DHA may prove beneficial as a non-pharmaceutical anti-inflammatory agent.

## Competing interests

Financial support for this work was provided in part by Minami Nutrition. The authors or the University of Memphis do not have any direct or indirect interest in EPA/DHA or Minami Nutrition.

## Authors' contributions

RJB was responsible for the study design, biochemical work, statistical analyses, and manuscript preparation; DL, AG, and KFW were responsible for data collection, blood collection and processing; BKS was responsible for the study design and manuscript preparation. All authors read and approved of the final manuscript.
